# Leptomeningeal Disease as a Rare Complication of Primary Penile Urethral Cancer

**DOI:** 10.1155/2020/6349456

**Published:** 2020-03-23

**Authors:** Nan Chen, Thomas Wheeler, Michael Coburn, Aihua Edward Yen

**Affiliations:** ^1^Baylor College of Medicine, Houston, TX, USA; ^2^Department of Hematology and Oncology, Baylor College of Medicine, USA; ^3^Department of Pathology, Baylor College of Medicine, USA; ^4^Department of Urology, Baylor College of Medicine, USA

## Abstract

Primary penile urethral cancer is a rare genitourinary malignancy arising from the epithelial lining of the urethra. Our patient is a 63-year-old male with newly diagnosed penile urethral carcinoma who presented with headache and was found to have leptomeningeal disease on imaging and cerebral spinal fluid analysis. He was treated with systemic and intrathecal chemotherapy with some response and improvement in symptoms. This is the first reported case of leptomeningeal disease as a complication of penile urethral carcinoma. Recognition and prompt treatment are important; however, overall prognosis of this entity remains poor.

## 1. Introduction

Primary PUC is a rare genitourinary malignancy arising from the epithelial lining of the urethra [1] and should be differentiated from urothelial cancer arising from the bladder as well as squamous carcinoma arising from the penile glans [2]. Urethral cancer histologically can be adenocarcinoma, squamous, or urothelial, and a retrospective study showed that many of these cancers can have squamous and urothelial histologic features [2]. Approximately one-third of patients, such as this case, are p16-positive, implicating HPV as a risk factor [2]. Up to half of patients will have a history of internal strictures, and 25% have a history of sexually transmitted diseases [3]. It is an aggressive malignancy, with a recently published large case series demonstrating a median overall survival of 21-49 months from diagnosis [2, 4]. African-American race was associated with increased mortality [4]. The most common sites of metastatic disease are the liver and lungs. This is the first reported case of leptomeningeal spread in a patient with primary penile urethral cancer.

## 2. Case Presentation

**Figure 1:**
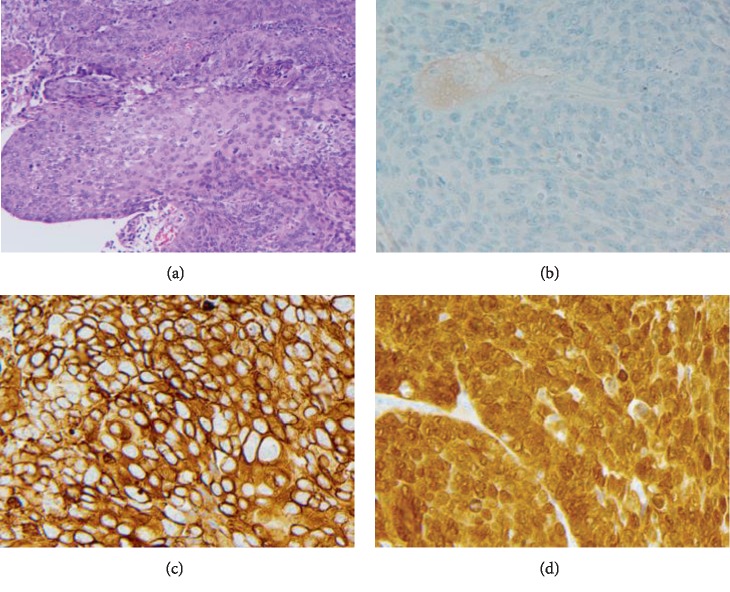
Stains of primary urethral tumor seen at 200x magnification. (a) Urethral squamous cell carcinoma hematoxylin and eosin stain. (b) Urethral squamous cell carcinoma with negative uroplakin stain. (c) Urethral squamous cell carcinoma with positive CK5/6 stain. (d) Urethral squamous cell carcinoma with positive p16 stain.

**Figure 2:**
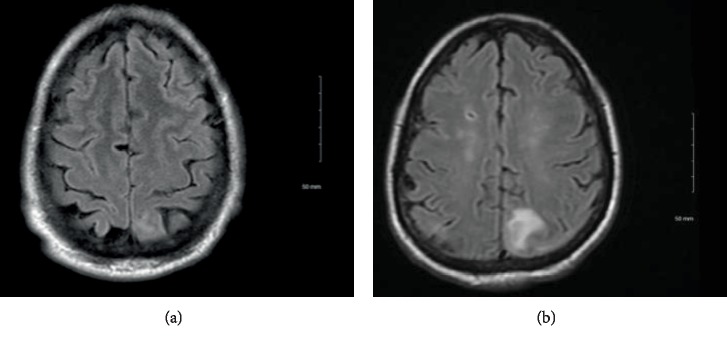
Magnetic resonance imaging of the brain showing progression of leptomeningeal disease. (a) Hyperintensity can be seen in L parasagittal sinus in this T2 Flair at time of diagnosis of LMD. (b) Increased hyperintensity in the same region in this T2 Flair taken 4 months after diagnosis of LMD.

The patient is a 63-year-old African-American man with a past medical history of hypertension, chronic kidney disease (grade II), and distal urethral stricture who presented with one year of hematuria during the beginning and end of his urinary stream. A cystoscopy noted a 7 mm ventral urethral lesion. A partial urethrectomy showed a squamous cell carcinoma of the penile urethra which was uroplakin-negative, CK5/6-positive, and p16-positive with carcinoma in situ in other areas (Figure 1). Staging computed tomography of his chest, abdomen, and pelvis showed pelvic and retroperitoneal lymphadenopathy, scattered lytic bone lesions, and soft tissue infiltration in the left femoral area consistent with metastatic disease. The patient was initiated on carboplatin/paclitaxel and completed 4 cycles complicated by grade 2 anemia and grade 1 peripheral neuropathy. Repeat imaging showed resolution of lymphadenopathy and stable lytic bone lesions. Given prior cytopenias and good response, he was continued with single-agent paclitaxel and then switched to nab-paclitaxel due to neuropathy. After his first cycle of nab-paclitaxel, the patient reported an intermittent occipital headache associated with double vision which improved when he closed one eye. Magnetic resonance imaging (MRI) of his brain showed a “faintly enhancing FLAIR signal abnormality in the parasagittal L superior parietal lobule” (Figure 2). Leptomeningeal disease was suspected, and MRIs of spine and lumbar puncture were pursued. Cervical, thoracic, and lumbar spine imaging was unremarkable, but lumbar puncture revealed cerebral spinal fluid (CSF) protein level of 56 mg/dL and cytology with poorly differentiated metastatic carcinoma. Systemic imaging did not show any progression of his disease. His course was complicated by seizures for which he was started on levetiracetam. An Ommaya reservoir was subsequently placed and intrathecal methotrexate was started twice weekly, corresponding to an improvement in headache and vision changes within two weeks. His CSF was negative for malignant cells after 6 weeks of treatment, and he continued on systemic pembrolizumab and weekly intrathecal methotrexate. While he demonstrated an initial clinical response, repeat MRI brain obtained 4 months after diagnosis showed progression of leptomeningeal disease (Figure 2). He developed worsening neurological symptoms, including headaches and altered mental status, and passed from his disease.

## 3. Discussion

Leptomeningeal disease is an underdiagnosed complication of numerous solid malignancies, most commonly breast, melanoma, and lung in which cancer cells invade the meningeal lining of the brain parenchyma [5]. A PubMed search found no other cases associating this complication with penile urethral carcinoma, and to the best of our knowledge, this case represents the first such report. Patients with LMD can present with a range of neurological symptoms ranging from altered mentation to seizures. If other sites of disease are present, treatment should consist of both anatomically directed and systemic therapy. A small proportion of patients, similar to ours, will experience LMD despite improvement in overall disease burden. This may be partially due to the fact that systemic therapy does not cross the blood-brain barrier, and the CSF becomes a haven for disease outside of the effects of systemic therapy [6]. This may also underlie the observation that LMD risk rises with longer cancer survival, which will likely occur with further improvement in cancer therapeutics [7]. LMD carries a very poor prognosis, and studies in the most common cancer types demonstrate overall survival of less than 6 months [8].

Intrathecal chemotherapy remains the mainstay of treatment. Agents which have been used in this context include methotrexate, cytarabine, and thiotepa [9]. Ommaya delivery has been shown to have improved overall survival compared to delivery via lumbar puncture and thus should be attempted when feasible [10]. Administration should be twice weekly until the CSF cytology becomes negative [5]. The role of radiation has not been definitively established, with retrospective studies suggesting it does not provide a survival benefit; however, it may be useful in patients with concomitant brain metastases or discrete, symptomatic lesions [7, 11]. Expert opinion currently favors a combination approach with intrathecal and systemic chemotherapy and radiation in certain cases [11]. With prompt recognition, patients can have improvement of symptoms and quality of life. Supportive care including steroids and antiepileptic drugs when needed should be considered in all patients with LMD. While our patient had initial symptomatic improvement with IT chemotherapy, his headaches returned within a few months, and imaging confirmed progression of leptomeningeal disease.

## 4. Conclusion

We present the first reported case of leptomeningeal metastasis from primary penile urethral carcinoma. Going forward, the suspicion for this complication must be considered even in patients with solid organ malignancies not commonly associated with a propensity to cause LMD, especially as novel cancer treatments continue to prolong survival. More effective treatment for LMD will be needed, as the prognosis for these patients remains poor. Our patient was treated with intrathecal chemotherapy and demonstrated symptomatic improvement.

## Additional Points

*Clinical Practice Points*. (1) Primary penile urethral cancer (PUC) is a rare and aggressive genitourinary malignancy arising from the epithelial lining of the urethra associated with poor prognosis. (2) We present the first case report of a patient with leptomeningeal disease (LMD) as a complication of penile urethral cancer. The suspicion for this complication must be considered even in patients with solid organ malignancies not commonly associated with a propensity to cause LMD, especially as novel cancer treatments continue to prolong survival.
